# Exploring the Non-Toxic Therapeutic Potential of *Dioscorea communis* in Combating Oral Pathogenic Bacteria and Their Effects on Hard and Soft Oral Tissues

**DOI:** 10.3390/biomedicines13040983

**Published:** 2025-04-17

**Authors:** Anastasia-Ioanna Papantonaki, Eleni Georgakopoulou, Christina Barda, Panagiota Loumou, Ioannis Sfiniadakis, Jane Anastassopoulou, Andreas Vitsos, Michail Christou Rallis

**Affiliations:** 1 Section of Pharmaceutical Technology, Department of Pharmacy, National and Kapodistrian University of Athens, Panepistimiopolis Zografou, 15784 Athens, Greece; tpapantonaki@gmail.com (A.-I.P.); eageorg@med.uoa.gr (E.G.); cbarda@pharm.uoa.gr (C.B.); ploumou@yahoo.com (P.L.); i.anastassopoulou@gmail.com (J.A.); avitsos@yahoo.gr (A.V.); 2 Athens Naval Hospital, Pathologoanatomic Laboratory, 11521 Athens, Greece; jsfiniadakis@yahoo.gr

**Keywords:** gingivitis, dental caries, ligature model, *Dioscorea communis*, phyto-therapeutics, in vivo

## Abstract

**Background/Objectives:** Gingivitis and dental caries are oral diseases resulting from bacterial accumulation in dental plaque, leading to inflammation, tissue destruction and the demineralization of tooth structures. *Dioscorea communis*, due to its anti-inflammatory and antimicrobial properties, could be a new treatment candidate. **Methods:** This study evaluated the preventive and therapeutic effect of a *D. communis* berry juice paste, formulated at 3% and 7% concentrations, on gingivitis and dental caries, in 55 male SKH-hr2 hairless mice. Gingivitis and dental caries were induced by ligation of the upper left incisor and the paste was applied topically three times daily, five days a week. Treatment efficacy was assessed through clinical examinations, photo-documentation, histopathological analysis and FT-IR spectroscopy. **Results/Conclusions:** Preventive administration of *D. communis* 7% significantly delayed disease onset, while therapeutic effects on established conditions were limited. Both concentrations were non-toxic to gingival tissues and dental structures.

## 1. Introduction

Oral diseases, including periodontal disease and dental caries, are highly prevalent conditions affecting diverse populations, from individuals with systemic diseases to the elderly suffering from xerostomia. Periodontal disease is characterized by gingival inflammation and the progressive destruction of the supporting structures of teeth. Gingivitis, its initial stage, is marked by reversible gum inflammation caused by bacterial accumulation and the host’s inflammatory response [1,2,3]. If left untreated, gingivitis can progress to periodontitis, where deeper inflammatory processes lead to periodontal ligament and alveolar bone destruction [4].

Periodontal diseases are primarily caused by bacteria, with different species playing a crucial role in their initiation and progression. The composition of dental plaque varies depending on its location; plaque adhering to the tooth surface mainly consists of Gram-positive rods and cocci, whereas plaque along the epithelial lining of the gingival crevice is predominantly composed of Gram-negative rods, filaments, and spirochetes. As subgingival plaque matures, certain bacterial genera become more prevalent, contributing to the development of periodontitis. These genera include *Treponema*, *Bacteroides*, *Porphyromonas*, *Prevotella*, *Capnocytophaga*, *Peptostreptococcus*, *Fusobacterium*, *Actinobacillus*, and *Eikenella*. Among them, *Porphyromonas gingivalis*, *Aggregatibacter actionmy-cetemcomitans*, and *Bacteroides forsythus* have been identified as the key pathogens associated with periodontitis [5,6].

Dental caries, a significant contributor to oral morbidity, result from acid production by the bacterial fermentation of sugars, leading to the demineralization of enamel. This condition disproportionately affects patients with systemic diseases, polypharmacy-induced xerostomia, and those with reduced manual dexterity who struggle with proper oral hygiene. The increased risk of caries and periodontal disease in these vulnerable populations underscores the necessity for innovative preventive and therapeutic strategies [7,8].

While fluoride remains the cornerstone of dental caries prevention, its widespread use is not without limitations, particularly in high-risk groups such as children, elderly individuals with reduced salivary flow, and patients with systemic conditions requiring alternative preventive solutions. The limited range of effective, clinically validated alternatives to fluoride highlights the urgent need for novel bioactive compounds with antimicrobial and remineralizing properties [9].

Preclinical models play a crucial role in advancing our understanding of oral disease pathogenesis and evaluating potential interventions. Various animal models, including the widely used ligature model, have been employed to induce and investigate periodontal disease [10]. This model, typically applied to molars, was adapted in this study for application to incisors, providing a reproducible and effective approach to simulating caries formation and periodontal inflammation in a controlled setting [11,12,13,14,15,16,17,18,19,20].

Natural products have emerged as promising candidates for dental therapeutics due to their broad pharmacological properties, including antimicrobial and anti-inflammatory activity [20]. The Dioscoreaceae family, comprising approximately 800 species distributed across tropical and subtropical regions, includes *Dioscorea communis* (L.) Caddick and Wilkin (syn. *Tamus communis* L.), a perennial climbing plant with a long history of traditional use [21]. *D. communis* is commonly found in regions of Europe, Asia, and North Africa, typically growing in temperate forests, woodlands, and rocky or mountainous areas. It thrives in well-drained soils and is often found at lower altitudes in these regions. The spherical to subglobose berries of *D. communis* have a mildly bitter and astringent taste and transform from green to a vivid, glossy red as they ripen, forming an alluring yet deceptive display, as these toxic fruits cannot be consumed without proper processing. It has been traditionally applied in folk medicine for treating musculoskeletal disorders, rheumatism, wounds, and bruises. The existing literature indicates that *D. communis* exhibits antioxidant, anti-inflammatory, and antimicrobial properties in both in vitro and in vivo studies [22]. Its aerial parts, particularly the berries, are rich in bioactive compounds that contribute to its pharmacological effects, such as amino acids, organic acids, lipids, terpenes–sterols, sugars, and phenolics [20,22]. The anti-inflammatory and antimicrobial properties of *D. communis* suggest its potential for managing periodontal disease and dental caries, especially in patients who require safe, effective, and fluoride-independent options [22,23,24,25,26]. A previous study demonstrated that *D. communis* berry juice extract exhibits significant antibacterial activity [22], providing evidence that its mechanism of action involves direct antibacterial effects.

The present study aimed to induce gingivitis and dental caries in a mouse model and assess the preventive and therapeutic efficacy of *D. communis* berry juice. By integrating ATR-FTIR spectroscopy as a non-invasive analytical method, this research provides novel insights into disease progression and treatment response, contributing to the broader effort of developing alternative strategies for oral disease management, particularly in high-risk and medically complex populations [27,28,29].

## 2. Materials and Methods

### 2.1. Animals and Study Design

Animal care was performed according to the guidelines established by the European Council Directive 2010/63/EU. The National Peripheral Veterinary Authority Animal Ethics Committee approved the experimental procedure (Protocol Numbers: 1566087/18-12-2023) after the affirmative opinion of the Animal Protocols Evaluation Committee.

Fifty-five male SKH-hr2 hairless mice, 4–5 months old, were used in this study. All mice originated from the breeding stock of the Small Animal Laboratory of the Section of Pharmaceutical Technology, Department of Pharmacy, National and Kapodistrian University of Athens. The animal room was kept at a temperature of 22–25 °C and humidity of 30–55% in a 12 h light–12 h dark cycle. The mice had unrestricted continuous access to food and fresh water.

For the induction of experimental gingivitis, a special diet was prepared. This diet included standard chow pellets (Nuevo SA-Farma-Efyra Industrial and Commercial SA, Oraiokastro, Greece), peanut butter (Smooth Peanut Butter, Healthy Habits, Thessaloníki, Greece), and water, in a ratio of 11:2:12, respectively.

The mice were divided into six groups: mice without ligation (normal), mice with ligation that did not receive any treatment (mice with ligation), mice with ligation that were preventively administered 3% *w*/*w* (preventive 3% *w*/*w*) and 7% *w*/*w* (preventive 7% *w*/*w*) *D. communis* berry juice, and mice with ligation that were therapeutically administered 3% *w*/*w* (therapeutic 3%) and 7% *w*/*w* (therapeutic 7%) *D. communis* berry juice. To reduce the number of animals used in the study, the control group without ligation was limited to 5 mice, while all other groups included 10 mice each. This decision was made because the clinical characteristics of normal mice are already well documented and consistent.

Throughout the experiment, the body weight of all mice was measured every 7 days, using a two-decimal balance, (KERN, PCB (Kern, Germany)), as an indicator of their physiological status and well-being.

**Figure 1:**
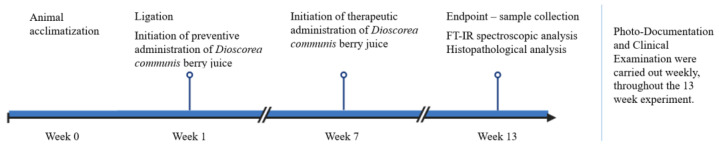
Schematic representation of the study design. This figure illustrates the experimental setup, including the treatment groups, time points for intervention, and methods of sample collection.

Figure 1 illustrates a schematic representation of the study design.

### 2.2. Plant Material, Paste Preparation and Administration

The aerial parts of *D. communis* were collected from cultivated populations in Stamata, North Attica (Central Greece) (coordinates (WGS84): latitude: 38°08′22.3″ N; longitude: 23°53′09.9″ E), during the flowering stage in August 2023. The berries were blended into juice, which was left to ferment for 3 months. It was then mixed with water (q.s.), sorbitol F solution (CAS: 50-70-4, Sigma-Aldrich Chemie GmbH, Schnelldorf, Germany), sodium benzoate (CAS: 532-32-1, Sigma-Aldrich Chemie GmbH, Schnelldorf, Germany), titanium oxide (CAS: 13463-67-7, Sigma-Aldrich Chemie GmbH, Schnelldorf, Germany), carbomer (CAS: 195739-91-4 Farmalabor, Milan, Italy), tixosil 73 (CAS: 112926-00-8, Solvay, Maharashtra, India), and tixosil 43 (CAS: 112926-00-8, Solvay, India) (Table 1) to formulate a paste whose texture and viscosity allow adherence and retention at the application area. *D. communis* berry juice was formulated at the concentrations of 3 and 7% *w*/*w*. The 3% concentration of *D. communis* was chosen based on traditional ethnopharmacological practices, which suggested it as an effective level for use. Meanwhile, the 7% concentration was selected following preliminary toxicity tests that ensured safety, allowing for the evaluation of a higher concentration to assess potential enhanced effects while maintaining a safe range. It was formulated into a paste, with the aim of eventually incorporating it into toothpaste for clinical application. This formulation is intended to adhere to the periodontal tissues for a sufficient duration, enhancing its therapeutic efficacy.

The paste was applied on the gums and teeth of the mice three times a day, five days a week. It was observed that the paste remained on the gums for approximately 30 s.

In the case of preventive administration, application began at week 1, the day after ligation, whereas in the case of therapeutic administration, it began after all clinical signs of gingivitis, i.e., erythema, oedema, and bleeding on probing, had occurred (at approximately 7–9 weeks).

### 2.3. Ligature Placement

All animals in ligation groups were anesthetized by an intraperitoneal injection of 100 mg/kg ketamine (Richter Pharma AG, Wels, Austria) and 7 mg/kg xylazine (Vetoquinol SA, Lure, France). Experimental gingivitis and dental caries were induced by placement of dental floss (Curaprox DF 820 PTFE Dental Tape, Kriens, Switzerland), tied around the upper left incisor with a double knot. The contralateral incisor in each mouse was left unligated to serve as a control. In all animals, the ligature was inspected daily and replaced or repositioned if necessary, especially during the first week, but also throughout the entire experiment. Ligatures were kept in place for 13 weeks, after which the animals were euthanized.

### 2.4. Periodontal and Dental Assessment

#### 2.4.1. Photo-Documentation

Photographs of the oral cavity of the mice were taken every 7 days, using a Nikon D5100 digital camera equipped with an AF-S Micro Nikkor 60 mm f/2.8 G (Nikon, Tokyo, Japan), to monitor their clinical condition and diseases’ progression.

#### 2.4.2. Clinical Evaluation

All mice underwent daily macroscopic examinations and clinical assessments every 7 days, using a scoring system ranging from 0 to 3 (0 = normal; 1 = erythema; 2 = erythema and oedema; 3 = erythema, oedema, and bleeding on probing); the severity of gingivitis was recorded accordingly. Additionally, the presence of dental caries was evaluated.

### 2.5. Sample Collection

On week 13, before the sacrifice of the mice, the ligatures were removed and stored at −20 °C.

All mice were euthanized by cervical dislocation. Gingival tissue samples were collected from the area above the upper left incisor of each mouse. Three tissue samples per study group were used for FT-IR analysis and then all samples were immersed in 10% formaldehyde solution for histopathological analysis.

### 2.6. FT-IR Analysis

FT-IR spectra were recorded under dry conditions using silica gel (Merck, Darmstadt, Germany) with a 4300 handheld FT-IR spectrometer (Agilent Technologies, Penang, Malaysia) equipped with an attenuated total reflection (ATR) crystal, with a resolution of 4 cm^−1^ and 60 scans/spectrum.

### 2.7. Histopathological Analysis

Gingival tissue samples were immersed in a 10% formaldehyde solution (BDH, Havant, UK), embedded in paraffin wax and sectioned at 5 μm thickness. Histological preparations were stained using hematoxylin–eosin and photographed under a 100× magnification to evaluate the extent of periodontal inflammation.

### 2.8. Nuclear Magnetic Resonance (NMR) Spectroscopy

NMR spectroscopy was employed to characterize the chemical profiles of the extracts used in this study. NMR spectra were recorded using a Bruker DRX 400 (400 MHz for ^1^H-NMR, Bruker BioSpin, Bill Rica, MA, USA) at a controlled temperature of 298 K. Chemical shifts were reported in parts per million (ppm, δ) relative to the solvent signals: 3.31 ppm for ^1^H and 49.0 ppm for ^13^C in methanol-d_4_ (CD_3_OD), and 7.24 ppm for ^1^H and 77.0 ppm for CDCl_3_.

To ensure a comprehensive chemical analysis, 1D and 2D NMR experiments, including correlation spectroscopy (COSY) and heteronuclear single-quantum correlation (HSQC) were conducted using standard Bruker pulse programs. This approach facilitated the in-depth characterization of their chemical profiles and confirmed the presence of target compounds.

For additional validation, 1D and 2D NMR spectra of the BuOH, MeOH, and H_2_O residues were also measured (see Supplementary Material for details). The chemical comparison was achieved by comparing the obtained chemical shifts and coupling constants with previously investigated extracts, as all extracts analyzed in this study had been previously chemically characterized by Tsami et al. [22]. The current NMR analysis verified the similarity of the ^1^H-NMR fingerprint of each extract, ensuring the reliability and reproducibility of the chemical profiling data.

## 3. Results

### 3.1. Photo-Documentation

**Figure 2:**
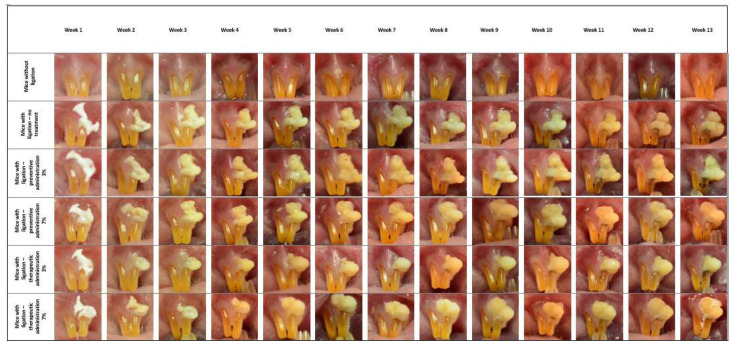
Representative digital images of the periodontium of mice from all experimental groups, captured weekly using a Nikon D5100 digital camera (Nikon, Tokyo, Japan). The images display the progressive changes over time.

As depicted in Figure 2, the presence of erythema begins at week 2 in all groups of the experiment. Symptoms intensify until week 5, when periodontal oedema is also present in all groups. From week 5 until week 13, the symptoms of gingivitis intensify, with the group receiving the preventive 7% administration exhibiting a slower progression of symptom severity. Regarding the mice that therapeutically received the preparation, at week 8, during which they exhibited all clinical signs of gingivitis, the application of the preparation began, but did not appear to lead to a significant reduction in symptoms until the completion of the experiment.

As far as dental caries are concerned, they first occur at week 5 in mice with ligation that did not receive any treatment and at week 6 in mice in the 3% and 7% preventive and therapeutic treatment groups.

### 3.2. Clinical Evaluation

**Figure 3:**
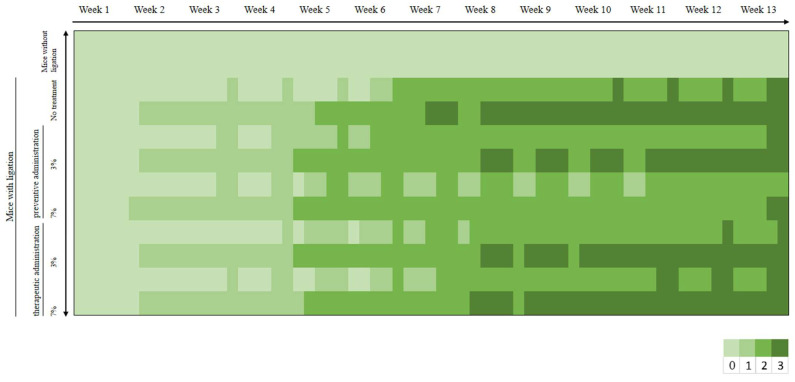
Heatmap illustrating the grading of each group according to the 0–3 scoring system utilized in the study. The scores assigned to each study group per week are color-coded, providing a visual representation of how the condition progressed over time within each experimental group.

Erythema had been observed in comparable percentages in all experimental groups since week 2, and by week 5, oedema also developed. In week 7, a small percentage of the mice with ligation that did not receive any treatment also exhibited bleeding on probing. Bleeding on probing was observed in mice in the 3% preventive treatment group and in those in the 3% and 7% therapeutic treatment groups at week 8. However, its appearance was delayed by 5 weeks in mice that received the 7% preventive treatment, occurring at week 13, with a lower incidence percentage compared to the other groups (Figure 3).

**Figure 4:**
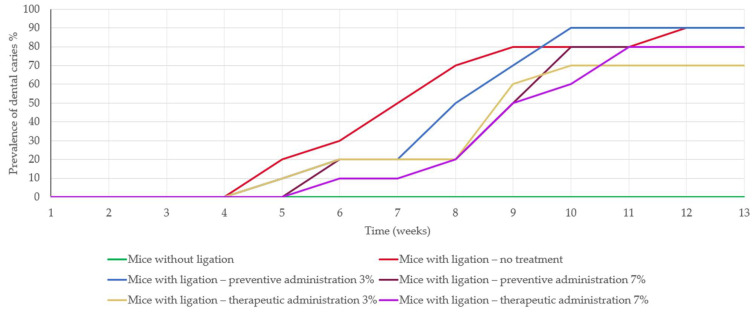
Diagram displaying the presence of dental caries. Each curve represents the percentage of dental caries presence in each experimental group.

Dental caries appeared at week 5 in 20% of the mice with ligation that did not receive any treatment. As for the mice in the preventive and therapeutic treatment groups receiving 3% treatment, 10% developed dental caries in the same week. However, the mice in the preventive and therapeutic treatment groups receiving 7% treatment exhibited dental caries one week later (Figure 4).

### 3.3. Histopathological Analysis

**Figure 5:**
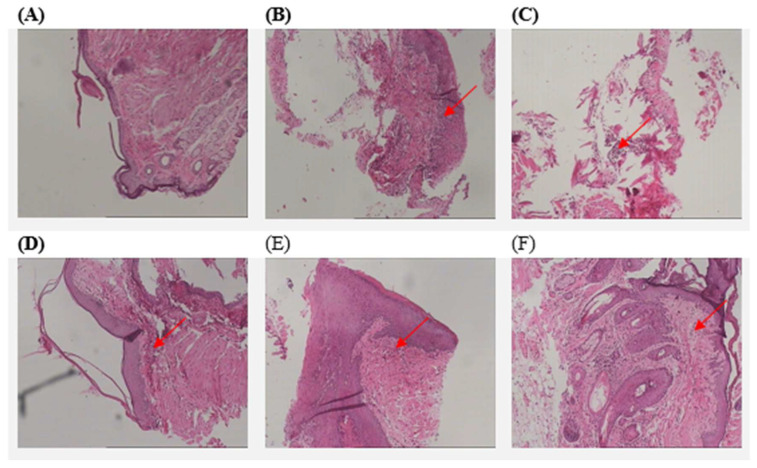
Representative histopathological images of the gingival tissue of all experimental groups (magnification 100×). Arrows pointing lymphocytes and polymorphonuclear leukocytes, indicative of inflammation. (**A**) Mice without ligation, (**B**) mice with ligation—no treatment, (**C**) mice with ligation—3% preventive administration, (**D**) mice with ligation—7% preventive administration, (**E**) mice with ligation—3% therapeutic administration, and (**F**) mice with ligation—7% therapeutic administration.

Histopathological images of mice in all study groups are depicted in Figure 5. Samples were stained with hematoxylin and eosin. Mice without ligation show the absence of inflammation. The histological picture of the mice with ligation that did not receive any treatment showed severe inflammatory infiltrations, characterized by the presence of lymphocytes and polymorphonuclear leukocytes. Samples of the 3% preventive treatment group show inflammatory infiltration, with varying intensities in different areas. The 7% preventive treatment group exhibits mild inflammation, with fewer lymphocytes and polymorphonuclear leukocytes. In both the 3% and 7% therapeutic treatment groups, moderate inflammation is detected.

### 3.4. FTIR Spectroscopic Analysis

ATR-FTIR spectroscopy provides a non-invasive, real-time method for obtaining detailed structural information on the skin barrier in vivo [27]. In this study, it generates a molecular fingerprint of the investigated skin area (periodontium). This technique enables monitoring of the specialized tissue surrounding the tooth. Through the measured spectra, the biochemical composition associated with lipids (*ca*3100–2800 cm^−1^, *ca*1740 and *ca*1450 cm^−1^), proteins (such as amide I and amide II; ca1650–1550 cm^−1^), water molecules, and glycoproteins (*ca*3000–2750 cm^−1^) [28] among others was identified, providing insights into the dynamic responses that occur in the presence of inflammation, bacterial increases, etc., during induced gingivitis.

**Figure 6:**
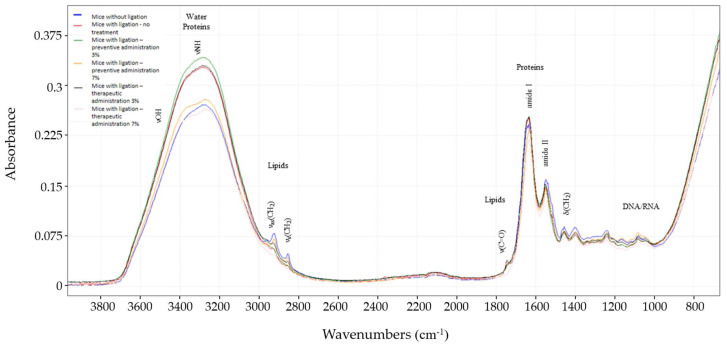
Comparison of normalized and averaged FTIR spectra of the gingival tissue samples, for the mice without ligation (blue), mice with ligation—no treatment (red), mice with ligation—3% preventive administration (green), mice with ligation—7% preventive administration (yellow), mice with ligation—3% therapeutic administration (black), and mice with ligation—7% therapeutic administration (pink). Changes in band frequency, intensity, and shape were observed across the spectral regions 3700–3000 cm^−1^, 3000–2850 cm^−1^ and 1800–800 cm^−1^.

As shown in Figure 6, starting from the 4000–3000 cm^−1^ region, related to water molecules around 3300 cm^−1^ (νOH), the intense bands may be attributed to the fact that the skin near the tooth in the periodontium is more hydrated. Nevertheless, normal mice show decreased levels. The same applies to the νNH of glycoproteins in this area (*ca*3300–3200 cm^−1^), where normal mice show decreased levels, while mice with ligation and no treatment group show increased bands. This may be attributed to the fact that glycoprotein expression increases in pathologies such as periodontal disease [29].

In the region around 3000–2800 cm^−1^, we can hypothesize that the skin of the periodontium of normal mice results in increased signals when compared to the other groups. As for the mice with ligation and no treatment group, the absorbance is lower and slightly shifted.

As this area is assigned to the asymmetric and symmetric stretching of CH_2_ (*νas*CH_2_ and *νs*CH_2_, respectively), it indicates major changes in lipid content and cell membranes. This phenomenon may be attributed to severe inflammation and the presence of gingivitis. It is also worth noting that the mice with ligation in the 7% preventive treatment group is very similar to the control, indicating the effect of the administrated paste.

The next band, at about 1745 cm^−1^, is assigned to the aldehyde (νCHO) group caused by lipid and protein peroxidations [30]. In normal mice, this peak appears higher compared to the rest of the groups, indicating a baseline level of aldehyde production and efficient management of oxidative stress. In the presence of inflammation, severe oxidative stress might lead to extensive damage and the breakdown of lipids and proteins. This could result in the consumption or further transformation of aldehyde groups into other oxidative products. Inflammatory processes can also upregulate enzymes and pathways that degrade aldehydes more rapidly, potentially reducing their accumulation and, consequently, their signal in the spectrum [31].

## 4. Discussion

Natural products have gained increasing interest in this regard, with *D. communis* emerging as a promising alternative due to its antimicrobial, anti-inflammatory, and non-toxic properties [22,23,24,25,26]. The present study demonstrated that *D. communis* berry juice paste, particularly at a concentration of 7%, exhibited a protective effect against both gingival inflammation and enamel demineralization, making it a strong candidate for broader application in oral disease prevention.

The ligature model employed in this study offers several advantages for studying periodontal disease and dental caries. The ligature model facilitates the accumulation and retention of a bacterial load in the periodontal area, leading to the induction of a localized inflammatory state within a short period of time [7]. Ligation, with the use of a Teflon dental floss, was performed on the upper left incisor using a reproducible procedure that does not require surgical intervention. The animals in the experiment were fed a soft food diet enriched with peanut butter to facilitate the adhesion of food to the periodontal area, resulting in bacterial accumulation.

Clinical evaluation (Figure 3) using a standardized scoring system revealed progressive gingival inflammation across all experimental groups that received ligation. This is consistent with ligature-induced experimental gingivitis, a method documented in the literature for its reproducibility, rapid onset, and ability to induce all clinical signs of gingivitis and periodontitis [11,12,13,19]. Preventive administration of *D. communis* in the higher concentration paste delayed the onset and reduced the severity of clinical signs compared to the untreated group. However, therapeutic administration did not demonstrate significant benefits once gingivitis was established, indicating potential limitations in treatment efficacy in advanced disease stages.

Moreover, the study demonstrated *D. communis*’s potential as a protective agent against microbial activity [32] and enamel demineralization leading to caries progression, particularly at a concentration of 7% (Figure 4). This aligns with previous research indicating the antimicrobial properties of various phytochemicals present in *D. communis*, such as flavonoids and tannins, which have been shown to inhibit bacterial adherence and biofilm formation [22,23,24,25,26,32]. Histopathological analysis of gingival tissues (Figure 5) further supported these findings, demonstrating varying degrees of inflammation among different treatment groups. More specifically, mice receiving preventive treatment with 7% *D. communis* paste exhibited the mildest histological changes, suggesting a protective effect at this concentration, whereas therapeutic administration groups presented moderate inflammation. The observed anti-inflammatory response [33] may be attributed to the presence of polyphenolic compounds in *D. communis*, which have been documented to modulate cytokine production and reduce oxidative stress in gingival tissues [20,22,33,34]. Compared to the severe inflammatory infiltrations observed in the untreated group, these findings highlight the impact of *D. communis* berry juice paste in reducing inflammation [33].

A key advantage of *D. communis* is its safety profile, as no toxicity was observed at either of the two concentrations studied [34]. Given its dual mechanism of action against both periodontal disease and dental caries, its application could extend beyond experimental models to real-world clinical use, particularly in populations where conventional chemical agents are less desirable due to their limitations [35]. Its potential incorporation into conventional toothpaste formulations, gels, or oral rinses would provide accessible options for daily use, making it particularly suitable for vulnerable populations, including the elderly [36], individuals with disabilities, and patients who rely on caregivers for oral hygiene [35,37].

For patients unable to perform mechanical plaque control, such as those in palliative care, individuals with severe neuromuscular conditions, or those recovering from extensive oral surgeries [35,37], a non-toxic, anti-inflammatory, and antimicrobial rinse or gel could serve as a practical alternative to brushing. This would allow both patients and caregivers to integrate an effective preventive strategy without the risks associated with fluoride ingestion or the challenges of mechanical cleaning [32,33,34,35].

This study pioneers the use of ATR-FTIR spectroscopy to capture spectral changes in the gingival area of mice (Figure 6), offering baseline data for understanding gingivitis. These findings lay the groundwork for real-time ATR-FTIR applications in dental research, potentially enhancing diagnostics and treatment monitoring, especially in pre-clinical studies.

While the study highlights the potential of natural products like *D. communis* in preventing periodontal disease and dental caries, translating preventive effects into therapeutic benefits remains a challenge. Further optimization of key parameters, including dosage, formulation to increase retention time at the application site, combination with other antimicrobial and anti-inflammatory agents, and timing of administration, may enhance its therapeutic efficacy in future studies.

## 5. Conclusions

In conclusion, this research provides valuable insights into experimental gingivitis and dental caries using a mouse model, highlighting the potential of *D. communis* as a preventive agent against these conditions. While preventive benefits were observed, optimizing therapeutic strategies for treating established periodontal disease and dental caries with natural product formulations remains an area for further investigation. Furthermore, the study demonstrates that the plant juice is practically safe even at a concentration of 7% and has the potential to be incorporated into toothpaste for the treatment of gingivitis and dental caries. This study contributes to the ongoing exploration of alternative treatments for oral health conditions, aiming to complement traditional therapeutic approaches with potentially safer and more effective natural agents. It should also be mentioned that a detailed evaluation of the antimicrobial potential, including the underlying mechanisms—whether through direct antibacterial action, modulation of the host immune response, or inhibition of bacterial adhesion—is crucial. The study design follows a standardized protocol suitable for investigating this topic in a mouse model. However, a key limitation is the contact time of *D. communis* paste with gingival tissues. Under the conditions of this model, the paste remained in contact with the affected gums for only 30 s. Moreover, no treatment applications were performed over weekends, which may have influenced the outcomes. To address these constraints, ongoing research is exploring the use of specific polymers to enhance contact time and improve the therapeutic efficacy of the formulation. Nevertheless, it is worth noting that this protocol not only induces gingivitis but also contributes to the development of caries. It successfully mimics their pathogenesis in humans, although it cannot replace clinical studies. Taken together, these findings support the continued exploration of *D. communis* as a promising natural agent for oral health applications, warranting further research to optimize its formulation and therapeutic potential.

## Figures and Tables

**Table 1:** *D. communis* berry juice paste formulation at 3% and 7% concentrations.

Trade name	INCI name	Function	%*w*/*w*
Sorbitol 70%	Sorbitol	Humectant	39.70
Sodium Benzoate	Sodium Benzoate	Preservative	0.50
Titanium Oxide	Titanium Oxide	Opacifier/Colorant	0.50
Carbopol Ultrez 10	Carbomer	Thickening Agent	1.00
Tixosil 73	Hydrated Silica	Abrasive/Thickening Agent	14.00
Tixosil 43	Hydrated Silica	Abrasive/Thickening Agent	8.00
*D. communis* berry juice			3 or 7
Water	Aqua	Solvent	q.s

## Data Availability

The original contributions presented in the study are included in the article and Supplementary Material, further inquiries can be directed to the corresponding author.

## Supplementary Materials

The following supporting information can be downloaded at: https://www.mdpi.com/article/10.3390/biomedicines13040983/s1, Figure S1: ^1^H-NMR chemical fingerprints of *Dioscorea communis* berry juice in MeOD.
