# Supplementary material for: Exploring the Non-Toxic Therapeutic Potential of Dioscorea communis in Combating Oral Pathogenic Bacteria and Their Effects on Hard and Soft Oral Tissues
Source: Biomedicines. 2025 Apr 17;13(4):983. doi: 10.3390/biomedicines13040983 (PMC12024764; doi:10.3390/biomedicines13040983)
Supplement: Supplementary file 1 [file biomedicines-13-00983-s001.zip › biomedicines-3520081-supplementary.pdf]

biomedicines

MDPI

Supporting Information

Exploring the Non-Toxic Therapeutic Potential of *Dioscorea communis* in Combating Oral Pathogenic Bacteria and Their Effects on Hard and Soft Oral Tissues

Anastasia-Ioanna Papantonaki [1], Eleni Georgakopoulou [1], Christina Barda [1], Panagiota Loumou [1], Ioannis Sfiniadakis [2], Jane Anastassopoulou [1], Andreas Vitsos [1] and Michail Christou Rallis [1,*]

[1] Section of Pharmaceutical Technology, Department of Pharmacy, National and Kapodistrian University of Athens, Panepistimiopolis Zografou, 15784 Athens, Greece; tpapantonaki@gmail.com (A.-I.P.); eageorg@med.uoa.gr (E.G.); cbarda@pharm.uoa.gr (C.B.); ploumou@yahoo.com (P.L.); i.anastassopoulou@gmail.com (J.A.); avitsos@yahoo.gr (A.V.)

[2] Athens Naval Hospital, Pathologoanatomic Laboratory, 11521 Athens, Greece; jsfiniadakis@yahoo.gr

* Correspondence: rallis@pharm.uoa.gr; Tel.: +30-2107274699

Metabolite Fingerprinting Based on ^{1}H-NMR Spectroscopy

Previous chemical investigation of the plant material under study, as detailed by Tsami et.al., (2022) [22], extensively characterized the phytochemical profile of the berry juice, identifying fatty acid esters, alkylamides, phenolic derivatives (with cichoric acid derivatives being predominant), and organic acids, notably rich in lactic acid. In the current study, freshly prepared berry juice was incorporated in the paste for *in vivo* evaluation of its therapeutic potential. To confirm the presence and stability of the juice's chemical composition, ^{1}H NMR chemical fingerprinting was performed. The analysis verified that the chemical profile remained intact, demonstrating that this raw material can be reliably utilized for further pharmacological and therapeutic applications (Figure S1).

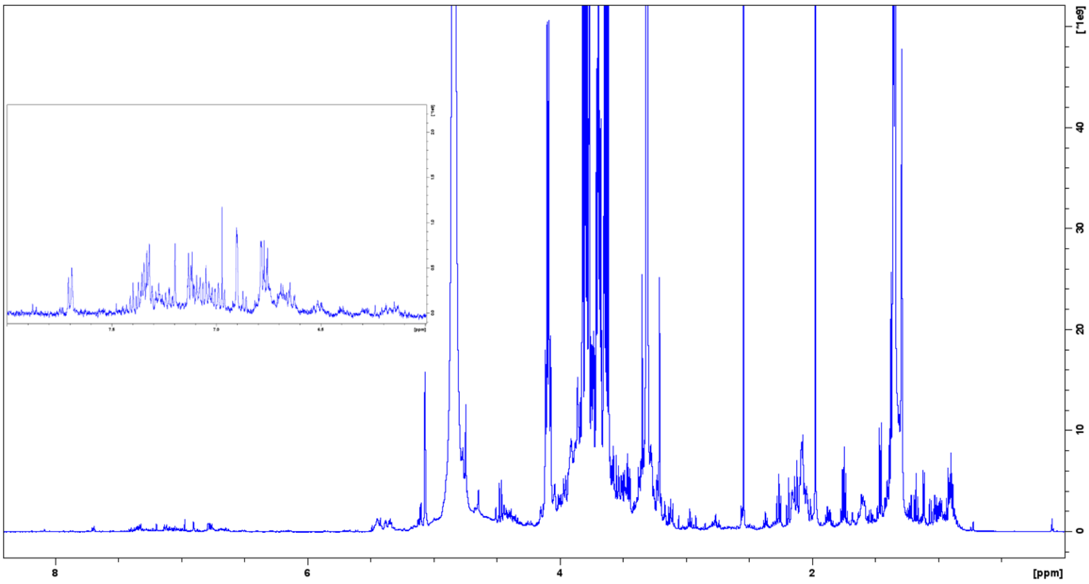

Figure S1. ^{1}H-NMR chemical fingerprints of Dioscorea communis berry juice in MeOD.

 https://doi.org/10.3390/biomedicines13040983
